# Long-term outcome in patients receiving permanent pacemaker implantation for atrioventricular block: Comparison of VDD and DDD pacing

**DOI:** 10.1097/MD.0000000000004668

**Published:** 2016-09-02

**Authors:** Jo-Nan Liao, Tze-Fan Chao, Ta-Chuan Tuan, Chi-Woon Kong, Shih-Ann Chen

**Affiliations:** a Division of Cardiology, Department of Medicine, Taipei Veterans General Hospital; b Institute of Clinical Medicine, and Cardiovascular Research Center, National Yang-Ming University; c Division of Cardiology, Taipei Municipal Gan-Dau Hospital; d Division of Cardiology, Department of Medicine, Cheng Hsin General Hospital, Taipei, Taiwan.

**Keywords:** dual chamber pacing mode, mortality, pacemaker, single lead atrial synchronous ventricular pacing mode

## Abstract

A permanent pacemaker (PPM) with dual chamber pacing (DDD) offers atrioventricular synchronization for patients with atrioventricular block (AVB). Single lead atrial synchronous ventricular pacing mode (VDD) is an alternative, but there are concerns about its efficacy and risk of atrial undersensing. Whether VDD can be a good alternative in patients with AVB remains unknown. The aim of the present study was to compare the long-term risk of mortality of VDD with DDD pacing.

A total of 207 patients undergoing PPM implantations for AVB with VDD mode were enrolled from 2000 to 2013. Another 828 age- and sex-matched patients undergoing DDD implantations during the same period of time were selected as the control group in a 1 to 4 ratio. The study endpoint was mortality.

A total of 1035 patients (64.3% male) were followed up for 46.5 ± 43.2 months. The mean ages were 75.0 years for VDD, and 74.9 years for DDD. The Kaplan–Meier survival analysis showed no significant difference in long-term survival between the VDD and DDD groups (log-rank *P* = 0.313). After adjustment for baseline characteristics, the VDD and DDD groups had a similar long-term prognosis with an adjusted hazard ratio of 0.875 (*P* = 0.445). Further analyses for the risk of cardiovascular and noncardiovascular deaths also showed no significant differences between the 2 groups.

The long-term prognosis of VDD mode is comparable to that of DDD mode. Single lead VDD can be considered as an alternative choice in patients with AVB without sinus nodal dysfunction.

## 1 Introduction

Permanent pacemakers (PPMs) are widely used for various types of symptomatic bradycardias. A pacing mode with preservation of atrioventricular (AV) synchrony is believed to be more physiological and beneficial based on major trials comparing single chamber atrial or ventricular pacing to dual chamber pacing (DDD).^[1]^ Therefore, the percentage of pacemakers using DDD keeps rising worldwide, especially in developed countries.^[2,3]^

For patients with atrioventricular block (AVB) and normal sinus node function, single lead atrial synchronous ventricular pacing mode (VDD) pacing, a physiological atrial synchronous ventricular pacing mode using a single lead with a floating dipole to detect atrial signals, can be used instead of DDD.^[4,5]^ However, available data about whether VDD is a good alternative to DDD are inconsistent.^[6,7]^ Several trials have shown a comparable efficacy of VDD and DDD pacing, but with a lower cost for VDD,^[6,8–10]^ while others demonstrated that up to one-third of patients with VDD failed to maintain AV synchronous pacing after years of follow-up.^[11]^

The concerns about atrial under-sensing and occurrence of sinus node dysfunction have impeded the use of VDD. However, most previous studies focused on the reliability of the detection of atrial signals by VDD systems, and data on patient outcomes after PPM implantations with VDD or DDD are limited. The aim of the present study is to compare the impact of different pacing modes, VDD versus DDD, on long-term survival of patients receiving PPM for AVB.

## 2 Methods

### 2.1 Study population

**Figure 1:**
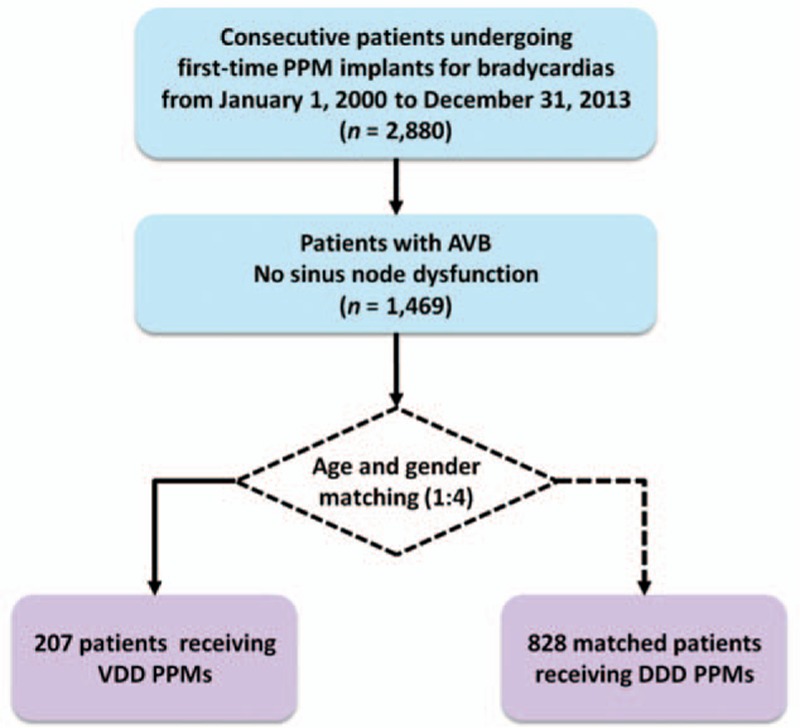
Flow diagram of study enrolment. From 2000 to 2013, a total of 207 patients with AVB undergoing PPM implantation with VDD mode were identified. Another 828 age- and sex-matched patients with AVB undergoing DDD mode implantation during the same period of time were selected as the control group at a 1 to 4 ratio. AVB = atrioventricular block, DDD = dual chamber pacing, PPM = permanent pacemaker, VDD = single lead atrial synchronous ventricular pacing mode.

From 2000 to 2013, a total of 2880 consecutive patients undergoing 1st-time PPM implantation in our hospital, a tertiary medical center in Taipei, Taiwan, were reviewed. Among them, 1469 patients were identified to have AVB and no obvious sinus node dysfunction. The absence of sinus node disease was confirmed by noninvasive tests, either holter monitoring or continuous electrocardiography monitoring at intensive care unit for at least 24 hours. VDD mode was implanted for 207 patients and they were selected as the study group. For each study patient, 4 age- and sex-matched patients among the remaining patients were selected as the control group. The study was approved by the Institutional Review Board at Taipei Veterans General Hospital, Taipei, Taiwan. The flow chart of the enrolment of the study population is shown in Fig. 1.

### 2.2 Implantation technique

The details of the implantation procedures have been described in our previous publications.^[12–14]^ Briefly, the skin was prepared with beta-iodine and alcohol for sterilization of the insertion site. Local anesthesia with xylocaine was administered before skin cutting. New leads were inserted transvenously through the cephalic vein or the subclavian vein. Generators were placed subcutaneously above the greater pectoral muscle. After completion of the procedures, the wound was sutured and sand bag compression was applied for 4 hours to prevent hematoma formation.

### 2.3 Postimplantation follow-up and clinical endpoints

Wound care was done after implantation with a 3-day course of empirical intravenous antibiotics. After discharge, patients were followed up at the pacemaker outpatient clinical of our hospital 2 weeks after implantation and then every 3 to 6 months for the evaluation of PPM function.

The study endpoint was all-cause mortality. Cardiovascular death was diagnosed as any death with a definite cardiovascular cause or any death that was not clearly attributed to a noncardiovascular cause. The occurrence of mortality was ascertained by review of medical records at our hospital and linking our database with the National Death Registry through a unique, life-long personal identification number given to every Taiwan citizen.

### 2.4 Statistical analysis

Normally distributed continuous variables are presented as the mean value and standard deviation. Categorical variables are shown as proportions. Comparisons of the continuous variables were performed using an unpaired 2-tailed *t* test, and the nominal variables were compared by Chi-square analysis or Fisher exact test. The event-free survival curve was plotted via the Kaplan–Meier method with the statistical significance examined by the log-rank test. The risk of mortality was assessed using Cox regression analysis. Variables with a *P* value <0.1 in univariate analyses were selected for multivariate Cox model. All statistical significances were set at *P* < 0.05, and all statistical analyses were carried out with SPSS 17.0 (SPSS Inc., Chicago, IL, USA).

## 3 Results

### 3.1 Baseline characteristics

A total of 1035 patients (64.3% male) were followed up for 46.5 ± 43.2 months (median 29.0 months, 1st quartile 12.3 months, 3rd quartiles 73.5 months). The baseline characteristics are shown in Table 1. The mean age was 75.0 ± 11.6 years for the VDD group, and 74.9 ± 10.9 years for the DDD group (*P* = 0.884). Hypertension was the most prevalent comorbidity, present in 78.3% of the VDD group and 70.4% of the DDD group (*P* = 0.024). The VDD group had more patients with heart failure, coronary artery disease, myocardial infarction, previous stroke or transient ischemic attack (TIA), end-stage renal disease (ESRD), and malignancy, and had fewer cases of atrial fibrillation. More patients in the DDD group were taking clopidogrel, dabigatran, or amiodarone compared to the VDD group.

**Table 1:**
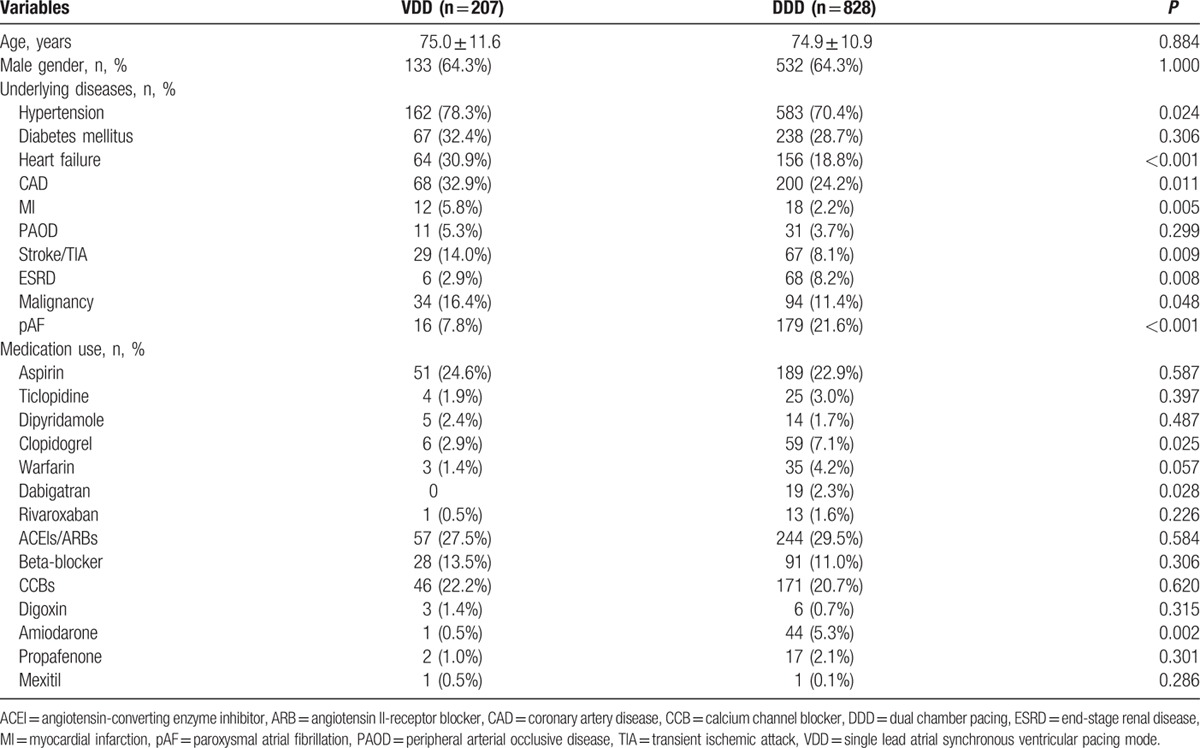
Baseline characteristics of subjects with VDD and DDD pacemaker.

### 3.2 Risk of mortality in VDD and DDD groups

**Figure 2:**
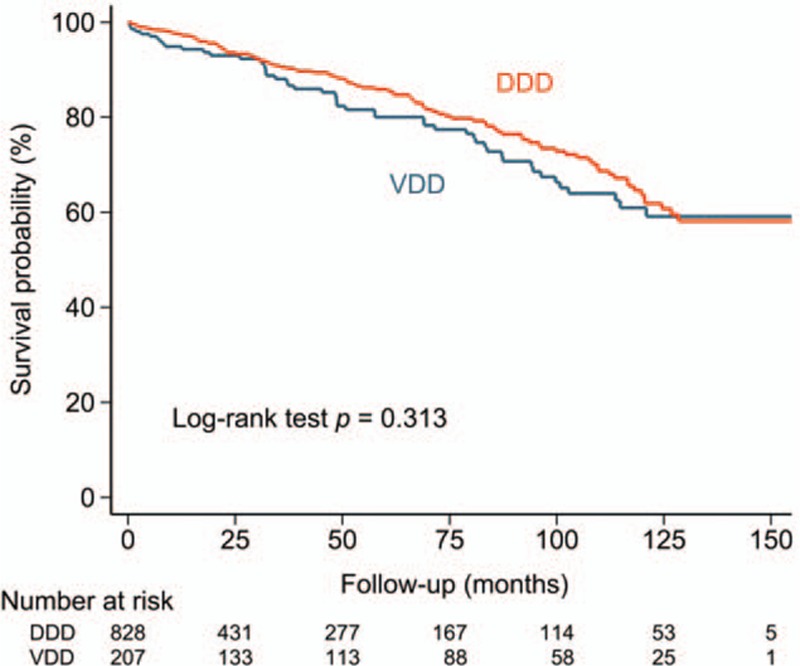
Cumulative risk of mortality of patients with AVB undergoing VDD or DDD implantations. During the follow-up, the risk of mortality did not differ significantly between the VDD and DDD groups. AVB = atrioventricular block, DDD = dual chamber pacing, VDD = single lead atrial synchronous ventricular pacing mode.

**Figure 3:**
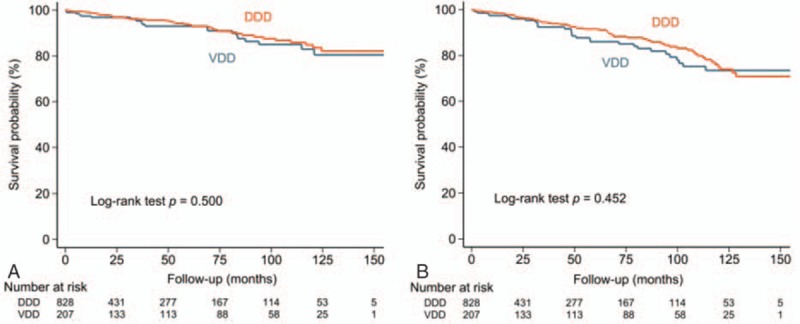
Cumulative risk of cardiovascular (A) and noncardiovascular (B) deaths of patients with AVB undergoing VDD or DDD implantation. The risks of cardiovascular (A) and noncardiovascular (B) deaths were similar between the 2 groups. AVB = atrioventricular block, DDD = dual chamber pacing, VDD = single lead atrial synchronous ventricular pacing mode.

During the follow-up, 50 patients in the VDD group and 110 patients in the DDD group died, with an annual mortality rate of 4.7% for VDD and 3.7% for DDD. The cumulative survival rate was not significantly different between the 2 groups as assessed with Kaplan–Meier analysis (Fig. 2, log rank *P* = 0.313). The annual risk of cardiovascular death was 1.9% for VDD, and 1.5% for DDD. The annual risk of noncardiovascular death was 2.8% for VDD, and 2.2% for DDD. The cumulative rates of cardiovascular and noncardiovascular deaths were not significantly different between the 2 groups (Fig. 3).

### 3.3 Risk factors associated with mortality

**Figure 4:**
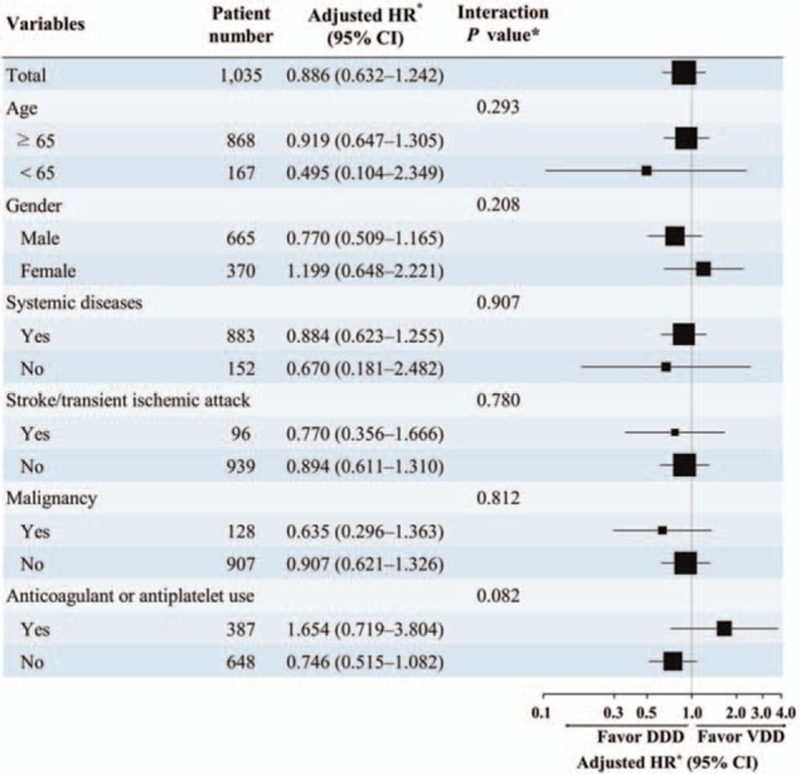
Pacing mode (VDD or DDD) and mortality risk in different subgroups of patients. The pacing mode, either VDD or DDD, did not significantly influence long-term prognosis in different groups of patients. Systemic diseases represent the presence of hypertension, diabetes mellitus, heart failure, end-stage renal disease, coronary artery disease, or myocardial infarction. DDD = dual chamber pacing, VDD = single lead atrial synchronous ventricular pacing mode

On univariate Cox regression analysis, heart failure, previous stroke/TIA, ESRD, and malignancy were associated with a higher risk of overall mortality, while pacing mode was not. After adjusting for heart failure, stroke/TIA, ESRD, and malignancy in a multivariable Cox regression analysis, pacing mode was also unrelated to mortality (hazard ratio = 0.875, 95% confidence interval = 0.621–1.233, *P* = 0.445), whereas heart failure, stroke/TIA, ESRD, and malignancy were independent predictors of mortality (Table 2). In the subgroup analysis, pacing mode did not influence overall mortality in different groups of patients (Fig. 4).

**Table 2:**
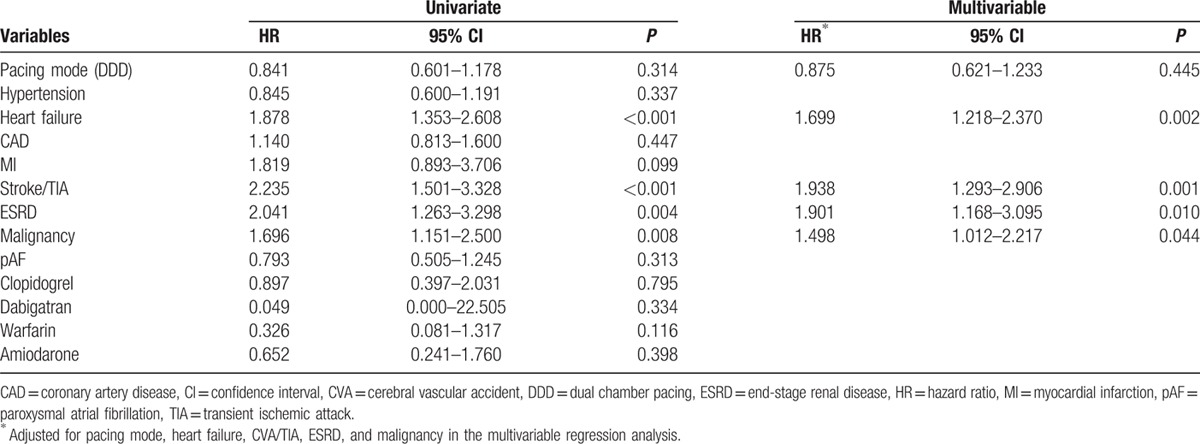
Risk factors in predicting overall mortality using Cox regression analysis.

## 4 Discussion

### 4.1 Main findings

In the present study, we compared the long-term survival rate in patients receiving VDD or DDD PPMs for AVB. Our principal findings were as follows: The risk of long-term mortality was similar for AVB patients receiving VDD or DDD PPMs; The presence of underlying diseases, including heart failure, stroke/TIA, ESRD, or malignancy, but not pacing mode, were important risk factors of all-cause mortality in patients receving PPMs for AVB.

### 4.2 Advantages and concerns of VDD mode

VDD pacemaker implantation is a physiological pacing mode with the advantages of shorter operation time, less fluoroscopic exposure, and lower complication rates as compared to DDD pacing.^[8,15,16]^ For aging people who usually have poor tolerance for operations, VDD can preserve AV synchronous pacing and the procedure may be easier to recover from.^[14]^ It is also a choice for AVB when the number of pacemaker leads needs to be limited, such as for those with small vascular access or with previously abandoned leads.^[1]^

However, atrial undersensing is one of the major concerns hindering extensive use of VDD mode. Undersensing is partly due to the floating dipole without contact with the atrial wall, which is prone to positional change, as well as local inflammatory changes leading to decreased atrial potential.^[17]^ The prevalence of atrial undersensing varied among different studies.^[8]^ Santini et al^[4]^ reported stable atrial signals over time in every physical activity, while extensive variation of atrial signal amplitude between postures was reported in another study.^[18]^ Marchandise et al^[19]^ found that up to one-third of VDD pacemaker patients could not maintain good atrial sensing after a mean follow-up of 2 years, which could mostly be ameliorated by increasing the atrial sensitivity. In the previous report from our group, inappropriate atrial sensing was noted in 16.6% of patients receiving VDD PPMs after a follow-up of 4.9 ± 2.5 years.^[13]^

In addition to atrial undersensing, the subsequent development of sinus nodal dysfunction after VDD implantation is also an important concern. Despite the potential risk of atrial undersensing and nonphysiological ventricular pacing for sick sinus syndrome, the results of the present study demonstrate that the long-term survival rates are comparable for patients receiving VDD and DDD PPMs. The risk of cardiovascular or noncardiovascular death did not differ significantly between the 2 modes. Therefore, the choice of pacing mode can be individualized, depending on the condition of each patient without considering the impact of different modes on long-term prognosis.

### 4.3 Clinical implications

Based on the findings of the present study, important comorbidities, not the pacing mode, were the major determinants of mortality for patients receiving pacemaker implantations for AVB. Our findings were consistent with that reported by Marchandise et al,^[19]^ showing that although a significantly larger number of VDD-paced patients developed poor atrial signal detection compared to DDD, the risk of mortality was similar. Compared to the study performed by Marchandise et al, the present study enrolled more patients to investigate this issue and may be able to lower the possibility of a type 2 error (false negative results due to limited sample size). Our findings suggest that controlling underlying diseases should be part of holistic management of patients after PPM implantation in addition to caring for the “device.”

### 4.4 Study limitations

There were several limitations in the present study. First, the variation of atrial signals was not reported, so the percentage of atrial undersensing is uncertain. Any adjustment of pacing mode after the 1st implant was also not recorded. Second, routine electrophysiology test was not performed in all patients with AVB and subtle sinus node abnormality could be neglected. Third, it is unknown how many patients developed sinus node dysfunction or atrial fibrillation after implantation. Fourth, we only focused on mortality and did not perform analysis for heart failure hospitalization or other common secondary outcomes. Fifth, the etiology and clinical course of AVB is quite complex in clinical practice and hence a diverse prognosis might be present. The cause of AVB was not identified in the present study and the result could be a mixture of various AVB. However, what we really focused on was the overall mortality between different pacing modes despite the variation of sensing signals, pacing percentage, or the alteration of pacing setting, which showed no difference after long-term follow-up. The finding was consistent even under subgroup analysis based on underlying characteristics or etiology of mortality. Finally, VDD patients had more comorbidities than DDD patients. However, we have tried to adjust these potential confounders in multivariate Cox models. Besides, despite more underlying diseases in VDD patients, it did not translate to a worse prognosis in long-term follow-up, meaning that even in fragile patients with AVB, VDD mode is probably feasible and provides equivalent long-term benefit. Therefore, the baseline differences between 2 groups may not significantly confound the results of the present study.

## 5 Conclusion

The long-term prognosis of VDD mode was comparable to DDD mode in patients with AVB. Risk of all-cause mortality depended on the presence of comorbidities, including heart failure, stroke/TIA, ESRD, or malignancy, but not the use of VDD or DDD. Therefore, single lead VDD can be considered as an effective alternative choice in selected patients with AVB.

## Acknowledgments

This work was supported in part by grants from the Ministry of Science and Technology (MOST 104-2314-B-075-024-MY3), and intramural grants from the Taipei Veterans General Hospital (V105B-023).
